# Essential Oils of Sage, Rosemary, and Bay Laurel Inhibit the Life Stages of Oomycete Pathogens Important in Aquaculture

**DOI:** 10.3390/plants10081676

**Published:** 2021-08-15

**Authors:** Anđela Miljanović, Dorotea Grbin, Dora Pavić, Maja Dent, Igor Jerković, Zvonimir Marijanović, Ana Bielen

**Affiliations:** 1 Faculty of Food Technology and Biotechnology, University of Zagreb, Pierottijeva 6, 10 000 Zagreb, Croatia; amiljanovic@pbf.hr (A.M.); dorotea.polo@gmail.com (D.G.); dpavic@pbf.hr (D.P.); maja.dent@pbf.unizg.hr (M.D.); 2 Faculty of Chemistry and Technology, University of Split, Ruđera Boškovića 35, 21 000 Split, Croatia; igor@ktf-split.hr (I.J.); zmarijanovic@ktf-split.hr (Z.M.)

**Keywords:** anti-oomycete activity, *Aphanomyces astaci*, EC_50_ values, Mediterranean wild plants, *Saprolegnia parasitica*

## Abstract

*Saprolegnia parasitica*, the causative agent of saprolegniosis in fish, and *Aphanomyces astaci*, the causative agent of crayfish plague, are oomycete pathogens that cause economic losses in aquaculture. Since toxic chemicals are currently used to control them, we aimed to investigate their inhibition by essential oils of sage, rosemary, and bay laurel as environmentally acceptable alternatives. Gas Chromatography–Mass Spectrometry (GC–MS) analysis showed that the essential oils tested were rich in bioactive volatiles, mainly monoterpenes. Mycelium and zoospores of *A. astaci* were more sensitive compared to those of *S. parasitica*, where only sage essential oil completely inhibited mycelial growth. EC_50_ values (i.e., concentrations of samples at which the growth was inhibited by 50%) for mycelial growth determined by the radial growth inhibition assay were 0.031–0.098 µL/mL for *A. astaci* and 0.040 µL/mL for *S. parasitica*. EC_50_ values determined by the zoospore germination inhibition assay were 0.007–0.049 µL/mL for *A. astaci* and 0.012–0.063 µL/mL for *S. parasitica*. The observed inhibition, most pronounced for sage essential oil, could be partly due to dominant constituents of the essential oils, such as camphor, but more likely resulted from a synergistic effect of multiple compounds. Our results may serve as a basis for in vivo experiments and the development of environmentally friendly methods to control oomycete pathogens in aquaculture.

## 1. Introduction

*Saprolegnia parasitica* and *Aphanomyces astaci* (Oomycota) cause significant economic losses in freshwater aquaculture: *S. parasitica* affects salmonid hatcheries and farms [1], and *A. astaci* crayfish rearing facilities [2,3,4]. *Saprolegnia parasitica* is parasitic to salmonid fishes such as *Oncorhynchus mykiss* and *Salmo salar* and infects all developmental stages [5,6,7]. It can also infect crayfish species, especially when the animals are injured, and its pathogenicity towards *Astacus astacus*, *Pacifastacus leniusculus*, and *Procambarus clarkii* has been confirmed by infection experiments [8]. *Saprolegnia*-infected fish develop a cottony mycelium on the gills and injured skin, while the infected eggs usually die as a result of hyphal rupture of the chorionic membrane and subsequent osmotic shock [9]. *Aphanomyces astaci* is a causative agent of crayfish plague. Its hyphae penetrate the cuticle of crayfish and spread throughout the tissues, leading to the development of a fatal disease [10,11]. It is mainly known for its destructive effect on native European crayfish species, while North American crayfish are considered carriers of the pathogen but can still succumb to the disease under certain stressful conditions, such as in a high-density farming environment [12,13]. Therefore, *A. astaci* may pose a serious threat to the crayfish farming industry on a global scale, both to native (e.g., *Astacus astacus*) and invasive (e.g., *Procambarus clarkii* and *Cherax quadricarinatus*) crayfish [2,4,14].

Diseases in freshwater aquaculture caused by oomycetes were previously treated successfully with malachite green, but its use is no longer allowed in the EU [15] and the USA [16] due to its teratogenic and carcinogenic properties [17,18,19]. Other chemicals currently used worldwide are also not sustainable solutions. In fact, formalin poses a health risk to fish farm workers who handle it [20] and to fish consumers, as it leaves residues in the fish [21]. Other agents such as bronopol, copper sulfate, and peracetic acid are less toxic but still pose a serious threat to the aquatic biota [22,23,24].

The toxicity of chemical agents used for oomycete control in fisheries prompts the search for environmentally friendly alternatives that should be effective against pathogenic oomycetes but also safe for operators, animals, and the environment. In this context, it has been increasingly shown that alcoholic extracts [25,26,27,28,29,30] and essential oils [31,32,33,34,35,36,37,38,39] of selected plants can inhibit pathogenic oomycetes in vitro, with essential oils generally being more potent than alcoholic extracts [38]. In particular, plants from the Lauraceae and Lamiaceae families, such as *Thymbra spicata* and *Cinnamomum zeylanicum*, have been reported to inhibit pathogenic freshwater oomycetes, mostly using the mycelium and zoospores of *Saprolegnia* spp. as models [32,34,36,37,38,40]. In comparison, the inhibitory activity of essential oils and their major components against *Aphanomyces* spp. is much less studied [28,40]. So far, there are no reports on the inhibitory activity of essential oils against *A. astaci*, but some essential oils showed good inhibitory activity against the phylogenetically related plant pathogen *A. euteiches* and the fish pathogen *A. invadans* [28,40].

The aim of this study was to test, for the first time, the inhibition of the oomycete pathogens *S. parasitica* and *A. astaci* by the essential oils of sage (*Salvia officinalis*), bay laurel (*Laurus nobilis*), and rosemary (*Rosmarinus officinalis*), which have previously shown potent antifungal and antimicrobial activity [41,42,43]. We analyzed the volatile composition of the essential oils and then tested the sensitivity of the alternative stages of the oomycete life cycle (i.e., mycelium and zoospores, as infectious stage) to essential oils.

## 2. Results

### 2.1. Chemical Composition of Essential Oils

**Figure 1:**
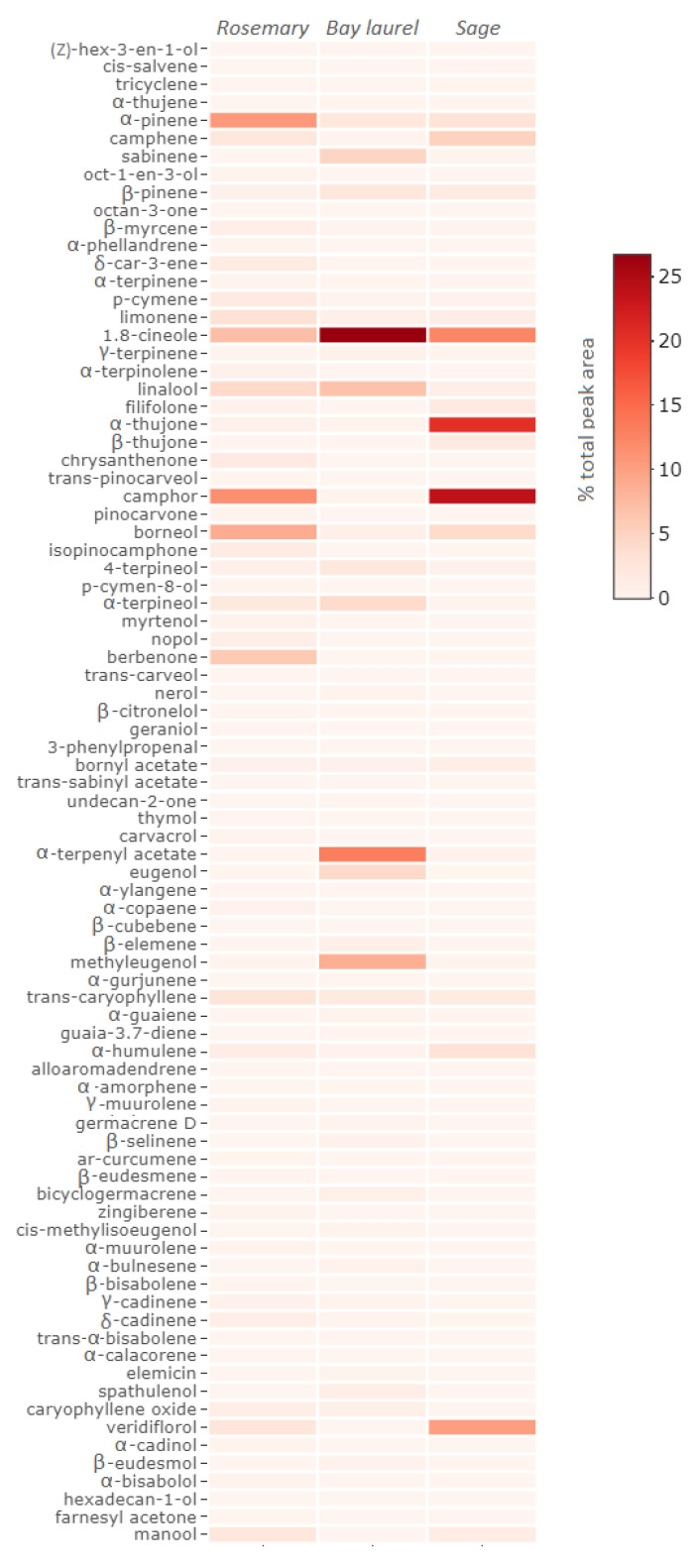
Heatmap of the volatile chemical composition of rosemary, bay laurel, and sage essential oils, as determined by Gas Chromatography–Mass Spectrometry (GC–MS).

The chemical composition of essential oils was analyzed by Gas Chromatography–Mass Spectrometry (GC–MS) (Figure 1, Table S1, Supplementary material). All essential oils were rich in volatile components, with the highest number of compounds identified in rosemary (65 compounds, 90% of total GC peak area), followed by bay laurel (53 compounds, 93% of total GC peak area), and eventually sage essential oil (35 compounds, 98% of total GC peak area). The major detected compounds in all three essential oils were monoterpenes, such as camphor (11.7%), α-pinene (10.8%), 1,8-cineole (7.3%), borneol (8.9%), and linalool (4.4%) in rosemary essential oil, then 1,8-cineole (26.8%), α-terphenyl acetate (13.2%), linalool (6.9%), sabinene (4.9%), and α-terpineol (4.1%) in bay laurel essential oil, and camphor (23.9%), α-thujone (20.3%), 1,8-cineole (12.5%), and camphene (5.2%) in sage essential oil. Some essential oils were also rich in sesquiterpenes, such as berbenone (6.1%), *trans*-caryophyllene (2.8%), and veridiflorol (2.6%) in rosemary essential oil, and veridiflorol (10.3%) and α-humulene (3.2%) in sage essential oil, while in bay laurel essential oil, a compound classified as a phenylpropane derivate, eugenol, was also identified in significant quantity (4.4% of total peak area).

### 2.2. Inhibition of Mycelial Growth

**Figure 2:**
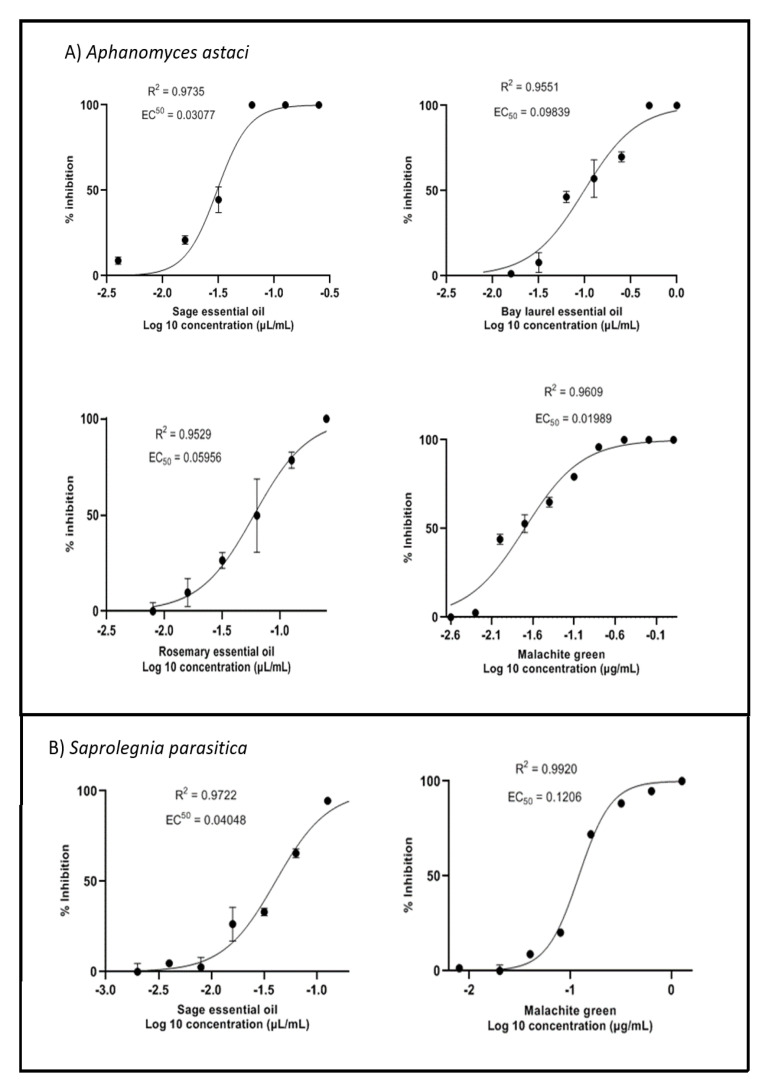
Mycelial growth inhibition curves of *A. astaci* and *S. parasitica* treated with sage, bay laurel, and rosemary essential oils. Non-linear regression curve fitting to estimate EC_50_ values (i.e., concentrations of samples at which mycelial growth was inhibited by 50%) for *S. parasitica* treated with rosemary and bay laurel essential oils could not be performed, since 100% inhibition was not achieved even with the highest tested concentrations. Mean values ± standard error (n = 3) are presented.

All essential oils tested inhibited mycelial growth of *A. astaci* (Figure 2, Table 1). Sage essential oil was the most effective, with an EC_50_ value (i.e., the concentration of the sample at which the mycelial growth was inhibited by 50%) two times lower than that of rosemary and three times lower than that of bay laurel (EC_50_ values were 0.03 µL/mL, 0.06 µL/mL, and 0.10 µL/mL, respectively). In the case of *S. parasitica*, only sage essential oil showed a significant inhibitory effect on mycelial growth (EC_50_ = 0.04 µL/mL), while rosemary and bay laurel essential oils showed no such effect, and 100% inhibition could not be achieved at the concentrations tested, so EC_50_ values could not be calculated (Figure 2, Table 1). Thus, the mycelium of *A. astaci* was more sensitive to essential oils of selected Mediterranean plants than the mycelium of *S. parasitica*: the EC_50_ value for the effect of sage essential oil on the mycelium of *S. parasitica* was higher than the corresponding EC_50_ value for *A. astaci* (0.04 vs. 0.03 µL/mL), while the effect of rosemary and bay laurel essential oils was significantly more pronounced towards *A. astaci* (data for *S. parasitica* not shown). Finally, the mycelium of *S. parasitica* was six times more resistant to malachite green (the EC_50_ value of malachite green for *A. astaci* was 0.02 µg/mL, while for *S. parasitica* it was 0.12 µg/mL).

### 2.3. Inhibition of Zoospore Germination

**Figure 3:**
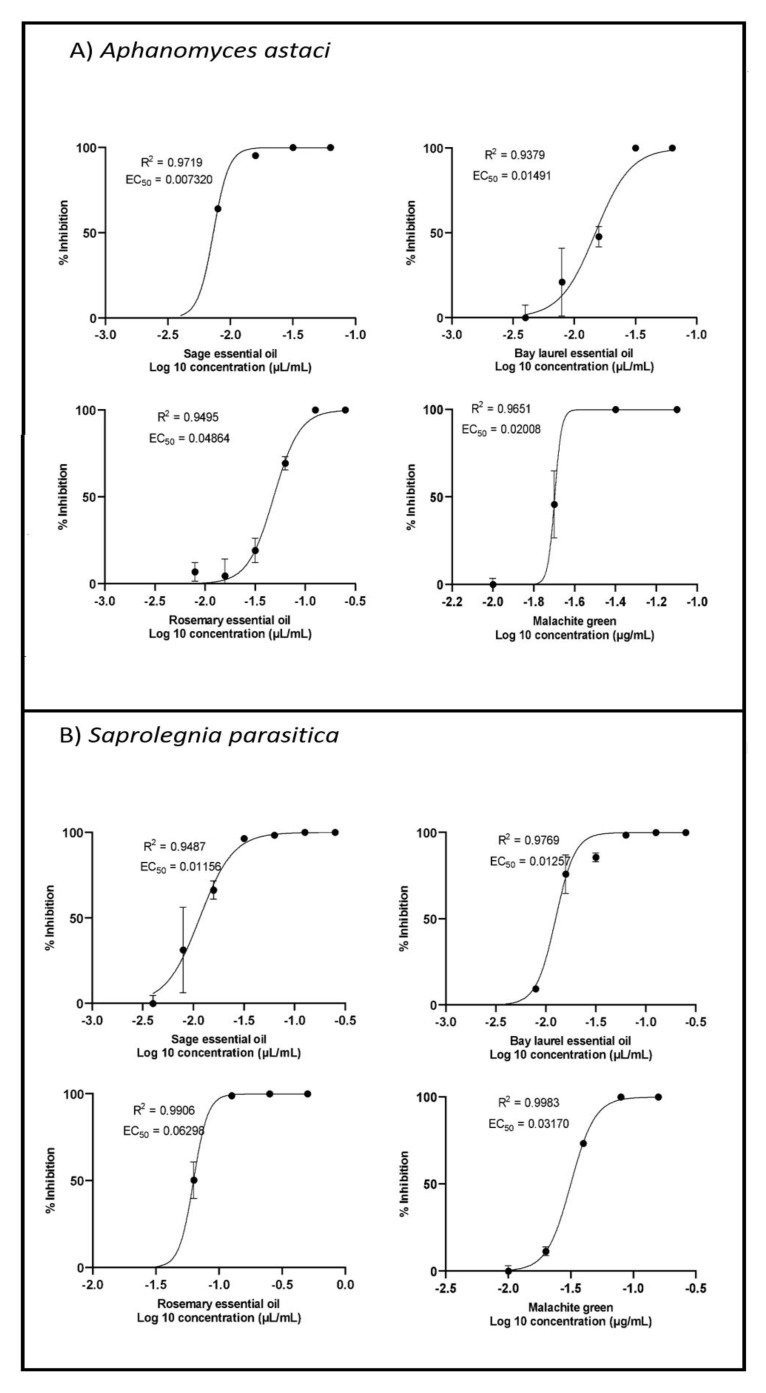
Zoospore germination inhibition curves of *A. astaci* and *S. parasitica* treated with sage, bay laurel, and rosemary essential oils. Mean values ± standard error (n = 3) are presented.

All essential oils inhibited the germination of zoospores of *A. astaci* and *S. parasitica*, with EC_50_ values ranging from 0.007 µL/mL to 0.063 µL/mL (Figure 3, Table 1). Among the essential oils tested, sage essential oil showed the strongest potency in inhibiting zoospore germination, followed by bay laurel essential oil and finally rosemary essential oil. In addition, the zoospores of *A. astaci* were slightly more sensitive to rosemary and sage essential oils (and malachite green) than the zoospores of *S. parasitica*, while the sensitivity of zoospores of both species to bay laurel essential oil was similar. When comparing the sensitivity of different life stages of pathogenic oomycetes, the zoospores were 4–6 times more sensitive than the mycelium to the effect of the tested samples. The only exception to this trend was the effect of rosemary essential oil and malachite green on the life stages of *A. astaci*, for which similar concentrations were required to inhibit zoospore germination and mycelial growth.

### 2.4. Correlation of the Observed Inhibitory Effects and Representation of Different Volatiles in the Essential Oils Studied

**Figure 4:**
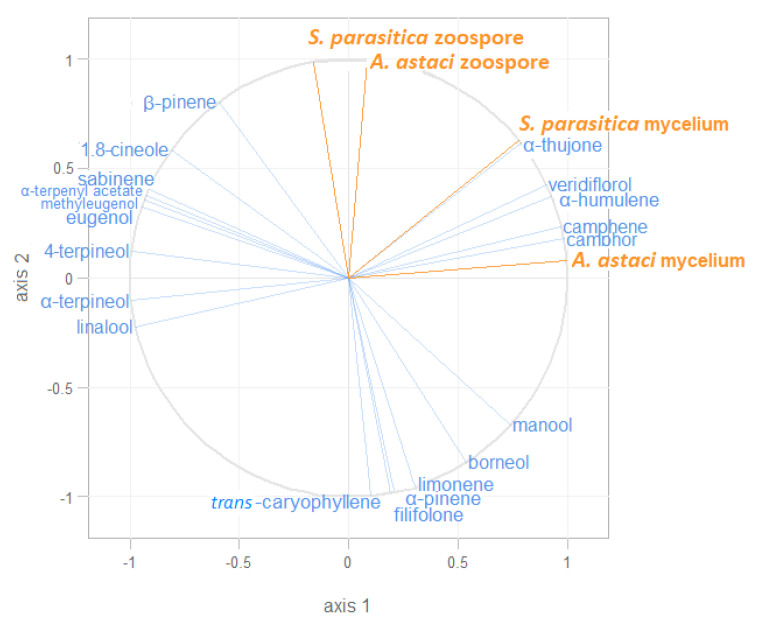
Partial least-squares regression (PLS-R2) radar of correlation. The orange lines represent the EC_50_ values for inhibition of mycelial growth and zoospore germination (response variables), while the blue lines represent volatile bioactive compounds present in the essential oils (predictors). Variables placed on the same side of the square within the circle are positively correlated, while those on the opposite side are negatively correlated.

The multivariate partial least-squares regression (PLS-R2) technique was used to investigate the relationship between the observed inhibitory effects (EC_50_ values for mycelial growth and zoospore inhibition) and the representation of different volatiles in the essential oils of sage, rosemary, and bay laurel. The relationship between the predictor variables (different volatiles) and the response variables (EC_50_ values) is visually represented in the form of a correlation radar (Figure 4). The results showed that camphor, camphene, α-humulene, α-thujone, and veridiflorol were positively correlated with the inhibition of mycelial growth and moderately correlated with the inhibition of zoospore germination of both pathogens. In addition, β-pinene and 1,8-cineole were positively correlated with the inhibition of zoospore germination of *S. parasitica*.

## 3. Discussion

We demonstrated for the first time the inhibitory potential of sage, bay laurel, and rosemary essential oils, as natural substances rich in volatile bioactive constituents, against mycelial growth and zoospore germination of two oomycete pathogens important in aquaculture, *S. parasitica* and *A. astaci*.

The essential oils tested showed significant inhibition of mycelial growth of *A. astaci* and, in the case of sage essential oil, inhibition of mycelial growth of *S. parasitica*. This is the first study to report the inhibitory effect of sage essential oil on the mycelium of *S. parasitica*, although the existing literature indicates the strong potential of plants from the Lamiaceae and Lauraceae families to inhibit the mycelial growth of *S. parasitica* [32,36,37,44]. However, it should be noted that previously reported inhibitory concentrations were up to three orders of magnitude higher than those determined in this study (i.e., 0.1–100 µL/mL compared to ~0.05 µL/mL). This may be partially explained by methodological differences, as the inhibitory concentrations for the disk diffusion assay (usually reported in the literature) are generally higher than the inhibitory concentrations for the agar dilution assay used here [45]. It is also possible that sage essential oil has stronger bioactive properties than previously tested plants from the Lamiaceae and Lauraceae families, such as oregano (*Origanum onites*), thyme (*Thymbra spicata*), savory (*Satureja cuneifolia*), and cinnamon (*Cinnamomum verum*) [32,36,44,46]. Moreover, this is the first report of the inhibitory effect of essential oils against *A. astaci*. Nevertheless, some authors reported a good inhibitory effect of tea tree oil against the mycelial growth of the fish pathogen *A. invadans* [28] and of 38 essential oils, including rosemary essential oil and essential oils from some other Lauraceae and Lamiaceae plants, against the mycelial growth of the plant pathogen *A. euteiches* [40].

Most of the existing studies focused on the inhibition of mycelial growth, while there are very few reports on the inhibition of zoospores, which are the infective stage [35,39]. Our study shows a good inhibitory activity of the tested essential oils against zoospore germination of *A. astaci* and *S. parasitica*, with EC_50_ values ranging from 0.007 to 0.063 µL/mL and sage essential oil being the most potent. Similarly, the essential oils of *Mentha longifolia* and *Thymus daenensis* (Lamiaceae) completely inhibited the germination of *S. parasitica* zoospores, but at much higher concentrations of 2.5 and 5 µL/mL, respectively [39]. This discrepancy can be partly explained by different protocols used but nevertheless indicates the promising properties of the essential oils tested here, especially sage.

We observed differences between species in sensitivity to the effects of the essential oils tested. The mycelium of *A. astaci* was much more sensitive than the mycelium *of S. parasitica*, and the same trend was observed for zoospores, although less pronounced. The higher sensitivity of mycelium and zoospores of *Aphanomyces* spp. compared to those of *Saprolegnia* spp. has been reported previously. Namely, an absolute ethanol extract of *Cassia fistula* (Fabaceae) inhibited mycelial growth of *S. parasitica* and *S. diclina* at 2000 µg/mL, compared to 500 µg/mL required for the inhibition of *A. invadans*, and similar values were obtained for zoospore germination [26].

The inhibitory effect of the essential oils tested differed markedly between oomycete life cycle stages, with zoospores being 1.2–6.5 times more sensitive than mycelium. For example, essential oils of bay laurel and rosemary showed strong inhibitory activity against zoospore germination of *S. parasitica* but had only a weak effect on mycelial growth. Previous studies comparing the sensitivity of mycelium and zoospores of pathogenic oomycetes to various compounds showed different results [26,27,35,47,48]. For example, the EC_50_ values of propamocarb hydrochloride for the inhibition of mycelial growth were two or more orders of magnitude higher than the EC_50_ values for the germination of zoospores of different isolates of *Phytophthora nicotianae* [47]. In another study, the concentrations of an absolute ethanol extract of *Cassia fistula* required to inhibit zoospore germination of *S. parasitica*, *S. diclina*, and *A. invadans* were similar to the concentrations required to inhibit mycelial growth [26]. Finally, the concentrations of *Laureliopsis philippiana* essential oil from bark and leaf required to inhibit zoospore formation of *S. parasitica* and *S. australis* were equal to or higher than the concentrations required to inhibit mycelial growth [35]. All of these studies, including the results presented here, suggest that some compounds are more potent zoospore inhibitors (such as the essential oils of sage, rosemary, and bay laurel), while others preferentially target the mycelium, or both life stages. This likely reflects differences in the mode of action of different compounds on zoospores and mycelium, as well as different detoxification mechanisms in different life stages of oomycetes, but further studies, including transcriptomic and proteomic analyses, are needed to clarify this.

The observed inhibitory activities of the essential oils could be attributed to their rich content of bioactive volatiles (mainly monoterpenes, such as camphor, and sesquiterpenes) and the synergistic activities of numerous minor compounds. The chemical composition of the tested essential oils was similar to those previously reported by our group [49] and in other studies [50,51,52,53,54]. Sage essential oil showed the strongest inhibitory potential on both pathogens and both life cycle stages. As indicated by PLS-R2 analysis, its anti-oomycete activity could be attributed to some of its major constituents, camphor, α-thujone, veridiflorol, camphene, and α-humulene, which were absent or present at low levels in other essential oils. In addition, β-pinene and 1,8-cineole (present in significant amounts in both sage and bay laurel essential oils) were positively correlated with the inhibition of *S. parasitica* zoospore germination, explaining the similar EC_50_ values of these two essential oils for *S. parasitica* zoospore germination (EC_50_ 0.012 and 0.013 µL/mL, respectively). Some of these compounds were previously reported to exhibit good anti-oomycete [44,55] and antifungal [56,57,58,59] activity. For example, camphor (up to 38.06 µg/mL) progressively slowed down the mycelial growth of *S. parasitica* and *S. delica*, while thujone and β-pinene (500 and 1000 µg/mL, respectively) inhibited the mycelial growth of *S. parasitica* [44,55]. Moreover, α-thujone and camphor have potent antifungal activity against *Fusarium graminearum*, *F. culmorum*, and *Schizosaccharomyces pombe*, which is mainly explained by the induction of oxidative stress and subsequent apoptotic cell death, but also by a decrease in genomic stability and epigenetic changes [56,57,58,59]. Thus, the high camphor content in sage essential oil probably contributed significantly to the observed inhibitory effects. The mechanism underlying the inhibition of oomycetes by camphor remains to be investigated but could be due to oxidative stress-mediated apoptosis, similar to the data obtained for fungal cells. However, it should be noted that it has previously been shown that the synergistic effect of many compounds present in essential oils is stronger than that of any single compound [35,44], and this was probably the case here.

Based on the obtained results, we propose that the essential oils of wild Mediterranean plants, especially sage, could be used as an ecologically acceptable method to control *A. astaci* and *S. parasitica* in aquaculture. This will require in vivo experiments to test the applicability of these essential oils either by dietary supplementation or by bathing the eggs and animals in essential oil suspensions. Previous studies suggest that the application of some essential oils and plant extracts may be useful in controlling *Saprolegnia* and *Aphanomyces* spp. infections in eggs and/or adults. For example, repeated incubation of *S. parasitica*-infected rainbow trout eggs with some essential oils, such as those of *Zataria multiflora* and *Satureja cuneifolia* at concentrations of 5 ppm or higher, resulted in an increase in hatching rate [34,36]. In addition, dietary supplementation with various essential oils, including sage essential oil and plant extracts, improved the immune response, fatty acid utilization, and growth performance of rainbow trout [60,61] and even conferred resistance to *A. invadans* infection in Indian major carp (*Labeo rohita*) [62]. In addition, immersion of infected fish in water containing 1% aqueous leaf extract of *Azadirachta indica* for 5 min daily for 24 days resulted in gradual healing of induced lesions in *Chana striata* [63]. However, some of the current constraints to large-scale application of essential oils, such as high extraction costs and variations in the composition of essential oils obtained from plants from different locations and seasons, have yet to be addressed. Production costs could be reduced by including inexpensive pretreatments prior to hydrodistillation that improve essential oils yields [49], while the quality of stock solutions could be standardized by optimizing growing conditions and harvest timing and by using genetic engineering [64,65]. Therefore, further experiments are needed as an extension of the results presented here to open the prospect of developing an ecologically acceptable control of *S. parasitica* and *A. astaci* infections through the application of essential oils. In particular, the possible toxicity of the effective concentrations of essential oils to fish/crayfish, as well as the possible rejection of the essential oil-supplemented feed due to their strong taste and odor [66], must be excluded.

## 4. Conclusions

The essential oils of Mediterranean wild plants are rich in bioactive volatiles, and in this study we demonstrated their potent activity against the pathogens *A. astaci* and *S. parasitica*. Thus, our results open a perspective for an environmentally friendly and sustainable control of diseases caused by oomycetes in salmonid and crayfish aquaculture through the application of essential oils. The inhibitory effect was particularly pronounced for sage essential oil, and some of its most abundant components, such as camphor, may be the major inhibitory molecules, although a synergistic effect of many minor components is also likely. Following our results, further studies are needed to develop protocols for the administration of essential oils as egg/animal baths or feed supplements and to standardize the production of essential oils on a large scale.

## 5. Materials and Methods

### 5.1. Microorganisms

Two oomycete pathogens of freshwater animals used were: *Aphanomyces astaci* (Schikora, 1906) strain B, PsI genotype (isolate PEC 8), and *Saprolegnia parasitica* Coker isolate CBS 223.65. *Aphanomyces astaci* was provided by F. Grandjean (University of Poitiers, Poitiers, France) and belonged to genotype PsI, which has been shown to exhibit marked virulence, particularly against native European crayfish species such as the noble crayfish *Astacus astacus* [67,68]. *Saprolegnia parasitica* CBS 223.65 is a reference strain isolated in the Netherlands from northern pike (*Esox lucius*) and was provided by R. Galuppi (University of Bologna, Bologna, Italy). Microorganisms were maintained in the laboratory at 18 °C on PG1 solid medium supplemented with ampicillin and oxolinic acid [69].

### 5.2. Plant Material and Essential Oil Isolation

Fresh leaves were collected from wild plants of rosemary (*Rosmarinus officinalis*), sage (*Salvia officinalis*), and bay laurel (*Laurus nobilis*) in the south Mediterranean region of Croatia in August 2020 and air-dried at room temperature (20 ± 2 °C) for one week. Dry leaves were packed in polyethylene bags and kept in a dark, dry, and cool place. Before being used for the extractions, all leaves were separated from branches and stems, and the bay laurel leaves were cut into four pieces each. The plant material was then ground using a household blender (Tefal) for 20 s into fine powder. The essential oils were isolated by hydrodistillation with reflux extraction pretreatment from 20 g of ground plant material mixed with 250 mL of purified water according to a previously developed protocol [49], yielding 0.2, 0.5, and 0.3 mL of oil/g of dry plant material of rosemary, sage, and bay laurel, respectively. For subsequent testing of anti-oomycete activity, the essential oils were diluted 1:9 in 96% ethanol. These 100 µL/mL stock solutions were then further diluted while performing the inhibition assays described in Section 5.4.

### 5.3. Gas Chromatography–Mass Spectrometry (GC-MS) Analysis

GC–MS analyses were carried out with an Agilent Technologies (Palo Alto, CA, USA) gas chromatograph model 7890 A equipped with a mass-selective detector (MSD) model 5975 C (Agilent Technologies, Palo Alto, CA, USA) and an HP-5MS 5% phenyl-methylpolysiloxane capillary column (30 m × 0.25 mm, 0.25 μm film thickness). In brief, the injector and detector temperatures were 250 °C and 300 °C, respectively; the column temperature was held at 70 °C for 2 min, was then increased from 70 to 200 °C at 3 °C/min, and was finally held at 200 °C for 18 min; 1.0 μL of the sample was injected using split mode (split ratio 1:50). Helium was used as a carrier gas (1.0 mL/min). The MSD (EI mode) was operated at 70 eV, and the scan range was set to 30–350 amu. Identification of volatile constituents was based on the comparison of their retention indices (RIs), determined relative to the retention times of a homologous series of *n*-alkanes (C_9_–C_25_), with those reported in the literature, and their mass spectra with those of authentic compounds available in our laboratories or those listed in NIST 17 (D-Gaithersburg, MD, USA) and Wiley W9N08 (Wiley, New York, NY, USA). Relative concentrations of components were calculated by the area normalization method without considering response factors.

### 5.4. Testing of Anti-Oomycete Activity of Essential Oils

#### 5.4.1. Inhibition of Mycelial Growth

The inhibitory effect of sage, bay laurel, and rosemary essential oils on mycelial growth of *S. parasitica* and *A. astaci* was tested using a radial growth inhibition assay followed by the determination of EC_50_ values (i.e., concentrations of samples at which mycelial growth is inhibited by 50%), as previously described [48], with some modifications. Malachite green, a known oomycete inhibitor [70], was used as a positive control (stock solution was 512 µg/mL in distilled water). Briefly, 100 µL of each sample was dissolved in 10 mL of molten PG1 medium and poured into 550 mm radius Petri dishes. In control plates, solvents were added in place of the samples, i.e., ethanol was used as a negative control for essential oils, and distilled water for malachite green. Up to eight twofold dilutions were tested for each sample, with initial concentrations of 1 µL/mL for essential oils and 1.28 µg/mL for malachite green. Each plate was inoculated by placing a 5 mm agar plug containing mycelium (taken from the edge of an actively growing mycelial mat) in the center and incubated at 18 °C. There was no statistically significant difference in the growth of the test species in the presence of ethanol or distilled water (*t*-test; *p* > 0.05). Three biological replicates were performed for each oomycete species, sample, and concentration. The assay was terminated after the mycelium in the negative control plates reached the end of the Petri dish, i.e., after five days for *A. astaci* and after two days for *S. parasitica*. Next, two perpendicular measurements of mycelial radius were taken for each plate and averaged, and the agar plug size was subtracted to obtain the final measurements of radial growth. Measurements from the sample plates were subtracted from the negative control measurements and converted to percent inhibition.

#### 5.4.2. Inhibition of Zoospore Germination

Sporulation of *A. astaci* and *S. parasitica* was induced by washing a grown mycelium incubated at 18 °C with sterile stream water, as described in detail in previous studies [67,71]. Zoospores were counted in the Thoma chamber using a light microscope (Zeiss Primo Star, Carl Zeiss, Oberkochen, Germany) at 100× magnification. The final concentration of zoospores was approximately 100,000 zoospores/mL for *A. astaci* and 80,000 zoospores/mL for *S. parasitica*.

To assess the effect of essential oils on the germination of zoospores of *A. astaci* and *S. parasitica*, we used previously described protocols with some modifications [48,71,72]. Up to six twofold dilutions of the test samples were used, with starting concentrations of 0.125 µL/mL for essential oils and 2.54 µg/mL for malachite green. To induce germination of *A. astaci* zoospores, 2 mL of stream water with the addition of CaCl_2_ (11.1 g/L) was mixed with 20 µL of the essential oil samples and 2 mL of water with zoospores and incubated in 12-well plates at room temperature for 16 h. In the case of *S. parasitica*, zoospores were vortexed for 45 s, then 2 mL of water with zoospore suspension was mixed with 2 mL of fresh PG1 liquid medium and 20 µL of essential oil samples and incubated for 1 h at 18 °C. After incubation, samples were photographed using an inverted microscope (Carl Zeiss, Oberkochen, Germany) at 200× magnification. Three replicates were made for each species, sample, and dilution. The percentage of germinating spores was determined by counting at least 200 spores in three to four randomly selected objective fields. A spore with a germ tube of at least one cyst diameter in length was counted as germinated. The germination percentage in the wells of negative controls to which solvents were added instead of samples was 33.4% and 97.5% for *A. astaci* and *S. parasitica*, respectively, in agreement with previously published data [71,72,73]. The germination percentages obtained for the different samples were converted to percent germination inhibition values.

### 5.5. Statistical Analysis

The heatmap of the volatile chemical composition of the essential oils of rosemary, bay laurel, and sage obtained by GC–MS was generated using the heatmaply package in R v. 3.2.0 [74] and the default methods for calculating the distance matrix (“euclidean”).

To estimate EC_50_ values for inhibition of mycelial growth and zoospore germination (i.e., concentrations of samples at which mycelial growth/zoospore germination was 50% inhibited), compound concentrations were log-transformed, data normalized, and nonlinear regression with curve fitting (by least squares) was performed using GraphPad Prism version 9.

To investigate the effects of different volatiles on the inhibitory potential of sage, rosemary, and bay laurel essential oils against *A. astaci* and *S. parasitica*, the partial least-squares regression (PLS-R2) approach was used. The response variables were the EC_50_ values for mycelial growth and zoospore germination, while the volatile bioactive compounds detected in the essential oils with at least 2% of the total GC–MS peak area served as predictors. PLS-R2 analysis was performed using the package “plsdepot” [75] in the program R v. 3.2.0 [74].

## Figures and Tables

**Table 1:** EC_50_ values for inhibition of mycelial growth and zoospore germination of *A. astaci* and *S. parasitica* by rosemary, sage, and bay laurel essential oils.

	EC_50_ for Mycelium Growth (µL/mL)		EC_50_ for Zoospore Germination (µL/mL)	
	*A. astaci*	*S. parasitica*	*A. astaci*	*S. parasitica*
**Rosemary essential oil** **Sage essential oil** **Bay laurel essential oil**	0.060	N.D. *	0.049	0.063
	0.031	0.040	0.007	0.012
	0.098	N.D. *	0.015	0.013
	**µg/mL**		**µg/mL**	
**Malachite green (pos. control)**	0.020	0.120	0.020	0.032

* N.D.: EC_50_ values could not be determined since 100% inhibition was not achieved with the highest concentration tested.

## Supplementary Materials

The following are available online at https://www.mdpi.com/article/10.3390/plants10081676/s1, Table S1: Volatile composition of essential oils determined by GC–MS.
