# Supplementary material for: Essential Oils of Sage, Rosemary, and Bay Laurel Inhibit the Life Stages of Oomycete Pathogens Important in Aquaculture
Source: Plants (Basel). 2021 Aug 15;10(8):1676. doi: 10.3390/plants10081676 (PMC8401702; doi:10.3390/plants10081676)
Supplement: Supplementary file 1 [file plants-10-01676-s001.zip › plants-1339859-SI.pdf]

Supplementary file 1 of the manuscript

Essential Oils of Sage, Rosemary and Bay Laurel Inhibit the Life Stages of Oomycete Pathogens Important in Aquaculture

Anđela Miljanović[1], Dorotea Grbin[1], Dora Pavić[1], Maja Dent[1], Igor Jerković[2], Zvonimir Marijanović[2], Ana Bielen[1,*]

[1] Faculty of Food Technology and Biotechnology, University of Zagreb, Pierottijeva 6, 10 000 Zagreb, Croatia; amiljanovic@pbf.hr; dorotea.polo@gmail.com; dpavic@pbf.hr; maja.dent@pbf.unizg.hr; abielen@pbf.hr

[2] Faculty of Chemistry and Technology, University of Split, Ruđera Boškovića 35, 21 000 Split, Croatia; igor@ktf-split.hr ; zmarijanovic@ktf-split.hr

* Correspondence: abielen@pbf.hr; Tel.: +385-1-483-6013

Table S1. Volatile composition of essential oils determined by GC-MS.

Compound	RI[1]	% total peak area		
		Rosemary	Bay laurel	Sage
(*Z*)-hex-3-en-1-ol	< 900	0.03	-	-
cis-salvene	< 900	-	-	0.14
tricyclene	929	0.16	-	0.20
α-thujene	932	0.03	0.23	0.08
α-pinene	941	10.79	2.48	2.96
camphene	956	2.41	0.33	5.15
sabinene	979	0.04	4.90	0.33
oct-1-en-3-ol	981	0.27	-	0.01
β-pinene	982	0.58	2.28	1.50
octan-3-one	990	0.15	-	-
β-myrcene	992	1.10	0.39	0.36
α-phellandrene	1010	0.25	0.06	0.04
δ-car-3-ene	1014	1.72	0.10	-
α-terpinene	1021	0.26	0.23	0.12
p-cymene	1030	1.82	0.36	0.49
limonene	1034	3.36	0.87	1.26
1.8-cineole	1038	7.29	26.79	12.53
γ-terpinene	1063	0.30	0.62	0.24
α-terpinolene	1091	0.65	0.10	0.18
linalool	1104	4.37	6.95	1.13
filifolone	1108	0.48	-	-
α-thujone	1110	0.80	0.37	20.34
β-thujone	1121	0.11	0.08	1.87
chrysanthenone	1131	1.78	-	-
trans-pinocarveol	1147	0.19	-	-
camphor	1149	11.71	0.39	23.86
pinocarvone	1169	0.33	-	-
borneol	1172	8.94	0.91	4.14
isopinocamphone	1181	1.67	-	-
4-terpineol	1182	1.02	2.25	0.61
p-cymen-8-ol	1191	0.27	-	-
α-terpineol	1195	2.05	4.12	0.34
myrtenol	1201	0.48	-	-
nopol	1209	1.12	-	-
berbenone	1212	6.11	-	0.23
trans-carveol	1224	0.09	-	-
nerol	1233	-	0.28	-
β-citronelol	1234	0.16	-	-
geraniol	1261	0.15	-	-
3-phenylpropenal	1275	-	0.06	-
bornyl acetate	1287	0.80	0.66	1.16
trans-sabinyl acetate	1294	-	-	0.07
undecan-2-one	1295	-	0.10	-
thymol	1296	0.11	-	-
carvacrol	1307	0.25	-	-
α-terpenyl acetate	1355	0.17	13.18	0.48
eugenol	1363	0.23	4.37	0.07
α-ylangene	1373	0.18	0.14	-
α-copaene	1378	0.52	0.05	-
β-cubebene	1391	-	0.03	-
β-elemene	1393	-	0.83	-

methyleugenol	1409	0.34	8.79	0.37
α-gurjunene	1413	-	0.10	-
trans-caryophyllene	1421	2.84	1.81	1.74
α-guaiene	1441	-	0.28	-
guaia-3.7-diene	1446	-	0.12	-
α-humulene	1456	1.24	0.40	3.17
alloaromadendrene	1462	0.04	0.17	0.18
α-amorphene	1478	-	0.07	-
γ-muurolene	1481	0.52	-	-
germacrene D	1482	-	0.28	-
β-selinene	1488	-	0.40	-
ar-curcumene	1489	0.07	-	-
β-eudesmene	1490	0.10	-	-
bicyclogermacrene	1496	-	0.91	-
zingiberene	1498	0.30	-	-
cis-methylisoeugenol	1500	-	0.25	-
α-muurolene	1504	0.25	-	-
α-bulnesene	1509	-	0.55	-
β-bisabolene	1513	0.19	-	-
γ-cadinene	1515	0.53	0.36	-
δ-cadinene	1525	1.17	0.34	0.07
trans-α-bisabolene	1545	-	0.14	-
α-calacorene	1546	0.13	-	-
elemicin	1561	-	0.33	-
spathulenol	1581	0.04	1.07	-
caryophyllene oxide	1584	1.18	0.84	-
veridiflorol	1594	2.57	-	10.31
α-cadinol	1646	0.27	-	-
β-eudesmol	1654	-	0.50	-
α-bisabolol	1689	0.27	-	-
hexadecan-1-ol	1886	0.06	-	-
farnesyl acetone	1924	0.19	-	-
manool	2055	2.25	-	1.42
Total %		89.6	92.2	97.2

[1]Retention index on HP-5MS
